# The Role of the S100 Protein Family in Glioma

**DOI:** 10.7150/jca.73365

**Published:** 2022-08-01

**Authors:** Haopeng Wang, Xiang Mao, Lei Ye, Hongwei Cheng, Xingliang Dai

**Affiliations:** Department of Neurosurgery, the First Affiliated Hospital of Anhui Medical University, Hefei, 230022, China.

**Keywords:** S100 protein, glioma, progression, diagnosis, prognosis

## Abstract

The S100 protein family consists of 25 members and share a common structure defined in part by the Ca^2+^ binding EF-hand motif. Multiple members' dysregulated expression is associated with progression, diagnosis and prognosis in a broad range of diseases, especially in tumors. They could exert wide range of functions both in intracellular and extracellular, including cell proliferation, cell differentiation, cell motility, enzyme activities, immune responses, cytoskeleton dynamics, Ca^2+^ homeostasis and angiogenesis. Gliomas are the most prevalent primary tumors of the brain and spinal cord with multiple subtypes that are diagnosed and classified based on histopathology. Up to now the role of several S100 proteins in gliomas have been explored. S100A8, S100A9 and S100B were highly expression in serum and may present as a marker correlated with survival and prognosis of glioma patients. Individual member was confirmed as a new regulator of glioma stem cells (GSCs) and a mediator of mesenchymal transition in glioblastoma (GBM). Additionally, several members up- or downregulation have been reported to involve in the development of glioma by interacting with signaling pathways and target proteins. Here we detail S100 proteins that are associated with glioma, and discuss their potential effects on progression, diagnosis and prognosis.

## S100 protein family: Types, structure and functions

**Figure 1:**
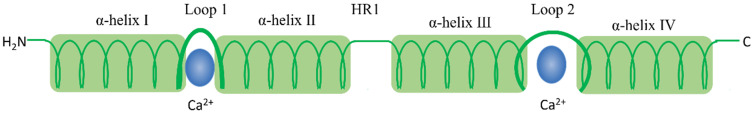
** S100 protein structural organization.** 1) S100 protein monomer is composed by EF-hand motifs, which contains two distinct different Ca^2+^-binding domains: 1) C terminal, characterized high Ca^2+^-binding affinity; 2) N terminal, has a weaker calcium affinity. Each monomer contains four α helical domains α-helix I, α-helix II, α-helix III, and α-helix IV. Helical loop 1 and loop 2 separate α-helix I and α-helix II, and α-helix III and α-helix IV, respectively. A flexible linker or hinge region (HR1) is also located between H-II and H-III.

**Figure 2:**
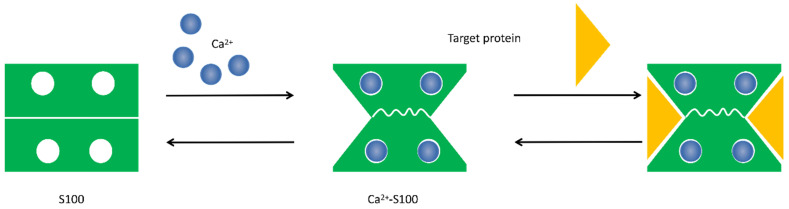
** Conformational change of S100 protein.** Ca^2+^ ion binds to the EF-hand motifs induce a conformational rearrangement, allowing the S100 protein to bind its cellular targets and regulate a large number of cellular functions.

The S100 protein family are low molecular weight and tissue/cell type specific proteins which are exclusively expression in vertebrates 1. At least 25 protein members have been identified since Moore first separated S100A1 and S100B from bovine brain in 1965 2, 3. Of these, encoding genes of 21 family members (S100A1-S100A18, trichohylin, filaggrin and repetin) distribute on chromosome locus 1q21, while other S100 proteins include S100P, S100Z, S100B and S100G locate at chromosome loci 4p16, 5q14, 21q22 and Xp22, respectively 4. Due to the constituents can be dissolved in ammonium sulfate solution at neutral pH, the subcellular fractions were termed as “S100”. These family of proteins are Ca^2+^-dependent proteins (and, except for S100A10, a Ca^2+^-independent protein), characterized by the presence of a complex grouping of EF-hand motif 5. EF-hand motif was first identified by Kretsinger and Nockolds in 1973, which comprises of two α-helices with an intervening 12-residue calcium-binding loop 6. Further structural analysis of S100 protein show that each of them has two different EF-hand-shape structure domains. Based on the above exceptional structure characteristics, S100 proteins can be exist as homodimers, heterodimers and oligomers, and perform distinct functions 4. To be specific, the two EF-hand motifs of each S100 protein contain two distinct different Ca^2+^-binding domains: C terminal (carboxy-terminal), composed of 12 amino acids, characterized high Ca^2+^-binding affinity; while N terminal (amino-terminal), formed by 14 residues, has a weaker calcium affinity. Additionally, a region between the two distinct EF-hand domains is called “hinge”, consists of 10-12 residues, which can increase the Ca^2+^-binding affinity 7. With Ca^2+^ ion binding to the EF-hand motifs, these proteins conformation will be rearranged, leading to hydrophobic regions exposed, which are thought the site of target protein binding. It is that Ca^2+^ interacts with S100 protein targets to regulate a large number of cellular functions 8 (Fig. 1 and Fig. 2).

## S100 proteins' functions in cancer

The physical and structural characteristics of S100 proteins indicate that they are trigger or activator proteins. Ca^2+^ binding is the way of most S100 protein families regulate structure and function, which allows them to change conformation that exposes hydrophobic regions in the molecules and act as Ca^2+^ sensors that can translate alterations in intracellular Ca^2+^ levels into a cellular response 9, 10. In addition to bind to Ca^2+^, individual S100 proteins also can bind to Zn^2+^, Cu^2+^ and Mn^2+^ to exert a series of intracellular and extracellular regulatory effects 11. Interestingly, although the majority of S100 protein are calcium-dependent, several calcium-independent S100 proteins such as S100A10 have been reported 4.

Based on the aforementioned processes and features, the functions of S100 proteins are diverse in cancer. Multiple members of the S100 family dysregulation are involved in tumor growth, apoptosis, differentiation, metastasis, angiogenesis and immune evasion *in vivo* 12, 13. Furthermore, extracellular S100 proteins interact with a variety of cell-surface receptors that initiate a cascade of signal transduction to realize these functions, including 1) receptor for advanced glycosylation end products (RAGE; also known as AGER), 2) G protein-coupled receptors, 3) Toll-like receptor 4 (TLR4), 4) scavenger receptors, 5) fibroblast growth factor receptor 1 (FGFR1), 6) CD166 antigen, 7) interleukin-10 receptor (IL-10R), 8) extracellular matrix metalloproteinase inducer (EMMPRIN; also known as basigin), 9) the bioactive sphingolipid ceramide 1-phosphate 12, 14-17. For example, S100A10, calcium-independent S100 protein, which is be identified as a novel biomarker in pancreatic ductal adenocarcinoma. The expression level of S100A10 mRNA and protein are significantly increased in human pancreatic tumors compared to normal ducts and nonductal stroma, and the knockdown of its expression could reduce surface plasminogen activation, invasiveness, and *in vivo* growth of pancreatic cancer cell lines 18. Recent studies reveal that S100A8/A9 mediated signaling through RAGE and TLR4 in activating specific downstream genes to promote tumor growth and metastasis 19, 20.

## S100 Proteins and Gliomas

**Figure 3:**
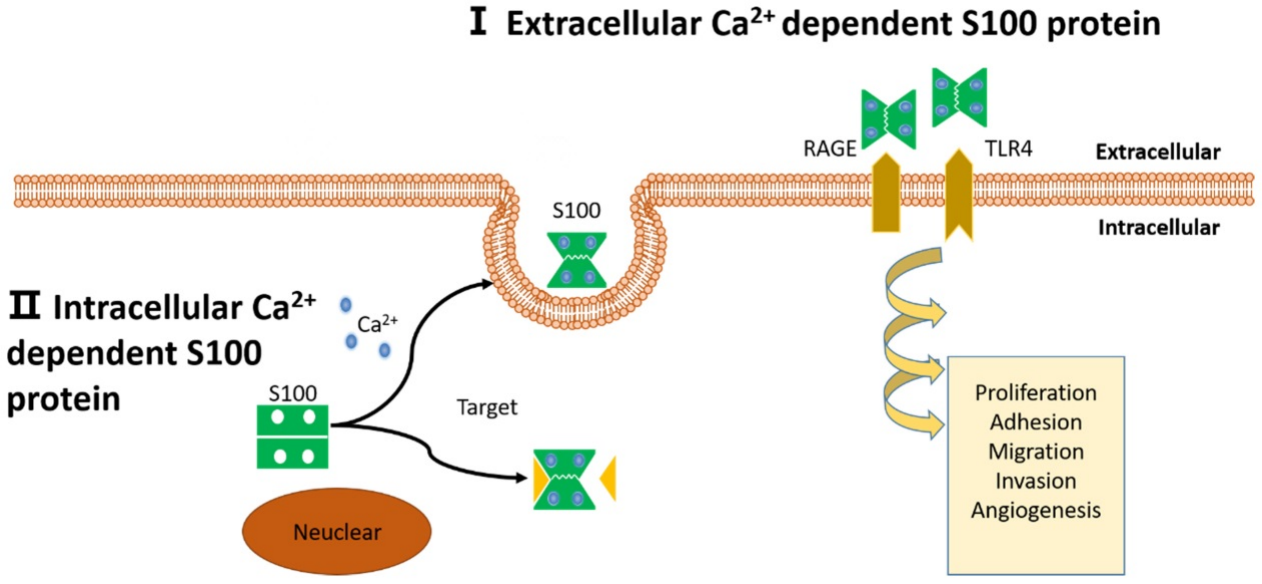
** Schematic representation of proposed intracellular and extracellular effects of S100 proteins.** (I): Extracellular Ca^2+^ dependent S100 proteins: S100 proteins interact with membrane surface receptors and activate a cascade of signaling responses to regulate pro-tumorigenic processes. (II): Intracellular Ca^2+^ independent S100 proteins: S100 proteins interact with target protein and activate a cascade of signaling responses or be secreted out of the cell.

Gliomas are the most common type of malignant brain tumors which are heterogeneous group of tumors developing from glial cells in the central nervous system. According to their histopathological characteristics, they are divided into high-grade and low-grade glioma. Especially high-grade glioma has an unfavorable prognosis with the median survival of only 12-15 months 21-23. Involving evidence indicates that S100 protein family are closely related to glioma in tumor progression, metastasis, invasion, and so on 24-27. Here, we reviewed the literature related to S100 proteins and their functions in gliomas (Fig. 3).

### S100A4

S100A4, also known as metastasin (Mts1), fibroblast-specific protein (FSP1), 18A2, pEL98, p9Ka, 42A, CAPL, and calvasculin, is localized in the nucleus, cytoplasm, and extracellular space. Its gene consists of four exons, of which the first two are noncoding 28, 29. Different to other S100 proteins, S100A4 has a quite long and very basic C-terminal loop following helix 4, which makes it particularly unique 29, 30. At molecular level, stimulating with Zn^2+^ induce its conformation changed that allows it to bind target proteins to enhance series of processes. Recent studies have demonstrated that S100A4 is directly involved in tumor metastasis and such as breast, non-small-cell lung, and glioma 30-33. In addition, it also has a certain correlation with cell survival, motility, invasion and epithelial-mesenchymal transition (EMT) 28, 29, 34.

S100A4 protein also plays an important role in glioma. An early study about the role of intracellular S100A4 for migration in rat astrocytes, which yielded some unexpected findings, S100A4 actually reduces the migratory capacity of white matter astrocytes 24. A further study indicated that low-grade glioma, which do not express S100A4, would be more incline to migrate along meninges and blood vessels, while S100A4 positive malignant glioma prefer to spread in areas of white matter. And this result showed that S100A4 may be an important factor in the pathogenesis of highly malignant brain tumor 35. However, Roberto Hernan et al. demonstrated that ERBB2 (HER-2/neu) may promote the metastasis of medulloblastoma via the up-regulation of S100A4 and several other prometastatic genes 33. In addition, a study by Kin-Hoe Chow et al. found that S100A4 expression is closely connected with tumorsphere formation and tumor initiating abilities *in vivo*. Selectively removing S100A4-expressing cells was able to sufficiently block tumor growth both *in vitro and in vivo*; and meanwhile, S100A4 is also identified as a critical regulator of glioma stem cells self-renewal both in mouse and patient-derived glioma tumorspheres in this research. Additionally, this study discovered that S100A4 acts as an upstream regulator of the master EMT in glioblastomas, due to it can regulate SNAIL2, ZEB and other mesenchymal transition regulators. Their research highlighted that S100A4 is not only a new biomarker and a regulator of glioma stem cells but also a mediator of mesenchymal transition and stemness in glioblastomas 36. S100A4 plays a significant role in some pathways as a mediator. A study by Ji Liang et al. showed that neutrophil-promoting malignant glioma progression was inhibited by S100A4 deregulation 37. Another study by Diana Aguilar-Morante et al. found that C/EBPβ (Enhancer Binding Protein β) could increase S100A4 levels by activating S100A4 promoter expression directly in murine GL261 and human T98G glioblastoma cells, and their data indicated that S100A4 play a crucial role of cell invasion and could mediate the observed effects of C/EBPβ on invasiveness of glioblastoma cells 25 (Table 1).

### S100A6

S100A6, also known as calcyclin, is located in the cytoplasm and nucleus in wide of cell types include adult normal tissues and several tumor cell types. Besides binding of Ca^2+^ with two EF-hand motifs, it also binds Zn^2+^ by not yet identified structures 38, 39. A large number of studies has proved that S100A6 is over-expressed in several diseases, especially in malignancies, such as non-small-cell lung cancer 40, gastric cancer 41 and pancreatic carcinoma 42. Recent studies have found that it is involved in the regulation of various cellular processes, including cell proliferation 41, cell apoptosis 43, migration 44, and so on. Moreover, several studies reported that S100A6 exert its extracellular or intracellular roles through interacting with binding or target proteins and activating the downstream signaling pathways 38, 45. For instance, study by Duan L et al. showed that a colorectal carcinoma cell line with relatively high S100A6 expression, leading to the inhibition of cell proliferation, migration and MAPK (mitogen-activated protein kinase) activity, further study suggest that S100A6 promotes the growth and migration by activating ERK1/2 (extracellular regulated protein kinase) and p38/MAPKs in colorectal carcinoma, and modulating of these pathways may be employed for colorectal carcinoma prevention and therapy 46. And another study indicated that S100A6 may promote nasopharyngeal carcinoma development via the activation of p38/MAPK signaling pathways and may be a new prognostic marker 47.

Dysregulated expression of S100A6 associates with glioma progression have been reported. A previous study has been shown to clearly distinguish between low-grade (WHO grade I and II) and high-grade (WHO grade III and IV) astrocytic tumors after altering the level of S100A6 protein expression 48. However, another study by Camby I et al. indicated that S100A6 is highly expressed in human astrocytic tumors, but this level of its expression cannot show a significantly functional change of the degree of tumor malignancy. Hence, it cannot be used as a discriminatory marker between the different grades 49. Furthermore, in ependymoma, a study by V Rand et al. have clearly proved that S100A6 is differentially expressed in ependymoma arising in different regions of the brain, and is significantly associated with supratentorial tumors 50. J C Lindsey et al. screened S100 genes for evidence of epigenetic regulation in medulloblastoma through a pharmacological expression reactivation approach, which found that S100A6 upregulated expression in multiple medulloblastoma cell lines after treatment with DNA methyltransferase inhibitor, 5′-aza-2′-deoxycytidine. Additionally, this study demonstrated S100A6 hypermethylation was significantly associated with the aggressive large cell/anaplastic morphophenotype 51. As mentioned earlier, overexpression of S100A6 is associated with cell motility in malignant tumor. The study by J Kucharczak et al. suggest that, gastrin could mediate cell motility in glioblastoma cells through activating gastrin-induced overexpression of the S100A6 gene product (tenascin-C) 52. Unfortunately, the mechanisms and signal pathways of S100A6 associated to tumor progression has, to present, not been studied in glioma.

### S100A8/9

S100A8 and S100A9, also called myeloid-related proteins (myeloid related protein 8 (MRP-8) and myeloid related protein 14 (MRP-14)) 53, 54. As the name suggest these proteins are specific highly expressed in myeloid cells, and a study indicated both the two proteins account for approximately 45% of cytosolic protein in neutrophils and approximately 5% in monocytes 54. Although S100A8 and S100A9 are able to form monomers, heterodimers, homodimers and trimers, it is reported that the S100A8/A9 heteromer (also known as calprotectin) is the clearly preferred complex due to its high stability 54, 55, And besides, they are frequently co-expressed and their expression is regulated together 54.

S100A8/S100A9 was original discovered as an immunogenic protein which was secreted by neutrophils with potent anti-microbial properties and then it is identified as a critical pro-inflammatory cytokine in acute and chronic inflammation 56, 57. Recently, researches have demonstrated that S100A8 and S100A9 dimers could interact with multiple receptors like RAGE (receptor for advanced glycation end products), TLR (toll-like receptor) and Wnt/β-catenin to promote tumor developments and invasiveness 58-60. In addition, S100A8 and S100A9 were reported highly expressed in tumors and primary expressed within tumors by immune cells. And their expression can induce the recruitment of myeloid cells and myeloid-derived suppressor cells resulted in tumor growth, metastasis, and formation of premetastatic niche 19, 61-64.

Recently, with rapid progress of proteomics, the identification of biomarkers are of great interest for early tumor growth, recurrence, and therapeutic response in oncology 65. A study by Anjali Arora et al. showed that S100A8 and S100A9 were highly expression both in the tissue and proteins in the serum, but only S100A8 presented a correlation with survival of GBM patients. Furthermore, this study found that S100A8 and S100A9 dependent on integrin signaling to promote migration and invasion at medium concentration 66. Similarly, the study of Paul R. Gielen et al. also found glioma patients have increased S100A8/9 serum levels, at the same time, this study confirmed that S100A8/9 protein expression was statistical significantly increased in myeloid-derived suppressor cells of patients with glioma 67. However, Gautam P et al. revealed that S100A9 levels was significantly elevated in individual plasma specimens from GBM patients 68. In addition, S100A8 and S100A9 are associated with progression and prognosis in glioma. The study by Jinsheng Xiong reported that S100A8 is significantly highly expression in glioma samples. Downregulated its expression inhibited cell proliferation, invasion, as well as migration of glioma. And circMAN2B2 could promote this biology processes by regulating the miR-1205/S100A8 axis 69. Furthermore, a previous study identified S100A8 and S100A9 involved neuron-to-astrocyte signaling process that may be important for astrocytoma formation, more specifically, the increased expression of S100A8 and S100A9 in neurons is an early and critical step in tumorigenic Kras-induced gliosis, which offers some important insights into their potential role in pre-cancer 70. There is growing evidence that GSCs are responsible for glioma formation and ongoing growth 71, 72. Studies of Song Chen et al. showed that S100A9 is highly expressed in GSCs with grade dependence and S100A9 regulates GSCs proliferation both *in vitro and in vivo* 73. Altogether, S100A8 and S100A9 play a significant role in proliferation, invasion, migration as well as glioma stem cell stemness via multiple target proteins and signal pathways. Detecting biomarkers of S100A8 and S100A9 in serum and tissue may be a diagnostic and prognostic maker.

### S100B

S100B, a Ca^2+^ binding peptide with a molecular weight of 20 kDA, existing as a homodimer composed of two beta subunits (EF-hand motifits). It is produced primarily by the astrocytes or spilled from damaged astrocytic cells and enter the bloodstream or extracellular space 5, 74. *In vitro and in vivo* experiments has been demonstrated it is involved in regulating cellular activities such as metabolism, motility and proliferation 75. S100B protein performs important functions in central nervous system (CNS) and is concentration-dependent. At low concentrations, it appears to exert neuroprotective effects against oxidative stress, but at high concentrations it produces neurodegenerative or apoptosis-inducing effects by increasing the expression of pro-inflammatory cytokines 26, 76, 77. S100B also over-expressed in tumors and modulated tumorigenesis via multiple signal pathways. For instance, S100B is elevated in primary malignant melanoma and interacts directly with p53, which are likely promoting tumor progression by excessive downregulating TP53 levels and activity 78. Additionally, S100B has been considered to contribute to tumorigenesis by regulate cell proliferation and differentiation by activating the mitogenic kinases Ndr 79 and Akt (protein kinase B) 80.

S100B is actively secreted by astrocytes or spilled from damaged astrocytic cells and could release into blood when the blood-brain barrier (BBB) is destroyed. Therefore, elevated serum level of S100B is a suggested marker in numerous nervous system diseases 81-83. Recently, several studies have indicated that serum levels of the S100B protein may be a valuable serum biomarker to predict the prognosis in glioma patients. The study by MAAIKE J. VOS et al. suggest that serum S100B might be a prognostic variable in cerebral glioma patients 84. However, F. K. Holla et al reported that while serum S100B seemed to have no prognostic value in newly diagnosed glioma patients, it may be valuable in terms of survival prognosis in patients with recurrent glioma 85. These findings are basically consistent with other studies 86, 87. Tumor-associated macrophages(TAMs) is an important component of inflammatory cells in tumor microenvironment and various chemokine are involved in TAM trafficking, and have been implicated in various aspects of cancer such as cell growth, angiogenesis and immunosuppression 88. A study demonstrated that over-expression of S100B in glioma promoted tumor growth by CCL2(C-C motif ligand 2) upregulation and TAM chemoattraction in murine models 89. Further study has identified Duloxetine, an S100B inhibitor, is able to shift TAM polarization into pro-inflammatory subtypes, which suggested that it may have anti-tumor properties 90. Furthermore, the S100B protein has been proposed to significantly contribute to glioma development by interacting with protein signaling pathways directly or indirectly. Flora Brozzi, et al. analyzed the effects of inhibition S100B expression in astrocytoma cell line GL15 and the Müller cell line MIO-M1 by knockdown S100B gene expression with small interference RNA technique, these results suggest that S100B might involve in the regulation of cell morphology, differentiation and migration through src-dependent activation of PI3K 27. Leying Zhang, et al. evaluated the effect of S100B-RAGE function on macrophages/microglia function in a murine glioma model, which found that glioma-mediated activation of STAT3 (signal transduction and activators of transcription 3) in macrophages/microglia might partly occur by the RAGE pathway and low levels of S100B induced STAT3 and inhibited microglia activation. Their findings suggest that the RAGE pathway may exert an important function in STAT3 induced glioma-associated macrophages/microglia, which may be mediated by S100B 91. In addition, a study reported that the S100B protein could reduce tumor suppressor p53 DNA binding and transcriptional activity, and meanwhile indicated that since S100B levels are significantly elevated in glioma and astrocytoma, it may contribute to glioma and astrocytoma progression by inhibiting p53 functions 92.

### S100P

S100P is a 95-amino-acid protein which was first purified and characterized from the placenta by Becker et al. in 1992. The “P” in its name indicates that it was purified first from placenta 93. There is increasing evidence suggesting that the deregulated expression of S100P associated with the tumor growth, progression and metastasis of various types of human cancer 94, such as breast 95, nasopharyngeal carcinoma 96 and pancreatic cancer 97. Evidence has been shown that S100P protein could mediate these processes by binding of Ca^2+^ ions, receptor for advanced glycation end products, cytoskeletal protein ezrin, calcyclin-binding protein/Siah-1-interacting protein and cathepsin D. Additionally, S100P could be applied as diagnostic marker, therapy target and prognostic/predictive indicator in a variety of different tumor types 94, 98.

Concerning to glioma, a study about immunocytochemical comparison of S100P, glial fibrillary acidic protein and vimentin in human glial tumors found that S100P was positive in most astroglial tumors and half of the oligodendrogliomas 99. Another study by Jennifer Nicole Sims et al. demonstrated that silence the expression of S100P dramatically inhibited cell proliferation, migration, invasion and anchorage independent growth in glioblastoma cells. At the same time, this study observed reduced cell migration, spheroid formation and expansion treated with di-ethylhexylphthalate (the most well-known phthalate) following S100P knockdown. Which suggested that S100P may be a potential therapeutic target and can be used as a biomarker for drug response 100. However, there have been few studies on S100P in glioma and the functional role of S100P and mechanisms in glioma has not been clearly elucidated until now.

### Other S100 proteins (S100A13, S100A16 and S100A11)

To our knowledge, these three proteins have not been extensively studied in glioma. One study investigated the relationship between the expression of S100A13 in human astroglioma in relation to tumor grade and vascularization, which results indicated that S100A13 is overexpressed in human high grade astrocytic gliomas and correlates with tumor grading and microvessel density 101. S100A16 is an astrocyte specific protein upregulate in tumors of different origins 102, Study by Szeliga M et al. found that transfection with liver-type glutaminase (LGA) cDNA increased the expression of LGA mRNA and protein and the ability of the cells to degrade glutamine, which result in reduction of survival, migration and proliferation of T98G glioma cells. And microarray analysis found decreased expression of S100A16 deserves attention in the context of LGA-induced phenotypic alterations 103. S100A11, also named S100C or calgizzarin, was found that played a significant role in GBM 104. Study by Tu et al. demonstrated that S100A11 plays key role in proliferation, EMT, migration, invasion and neurosphere formation in GBM cells and associated with poor survival of GBM patients 105. Another study by Yin-Hsun Feng et al found that Allopregnanolone could suppress GBM cell survival by decreasing DPYSL3 (dihydropyrimidinase-like-3)/S100A11 expression and inducing DNA damage 106.

## Conclusion and perspective

S100 proteins are frequently expression in gliomas and their dysregulated expression are closely associated with tumor progression, diagnosis and prognosis. In this review, we summarized current findings and progresses about S100 proteins in gliomas that have contributed to our understanding of the relationship between S100 proteins and gliomas. There is a considerable amount of literature on the contributions of S100 proteins to tumor progression, diagnosis and prognosis in glioma and its subtypes. However, the molecular mechanisms of S100 protein including S100A6, S100P, S100A13 and S100A16 is still unclear at present. As the research moves along, an increasing number of researches may reveal the underlying mechanisms S100 proteins in the progression of glioma and S100 protein will also act as an important prognosis marker and therapeutic target gradually.

## Figures and Tables

**Table 1:** Up- or downregulation and effects of S100 proteins in different subtype of gliomas

Types of S100 proteins	Subtypes of gliomas	Up- or downregulation	Functions	References
S100A4	Low grade gliomas	-	incline to migrate along meninges and blood vessels	35
	Malignant gliomas	↑	prefer to spread in areas of white matter	
	Medulloblastoma	↑	promote metastasis	33
	Glioblastoma	↑	a regulator of glioma stem cells and mediator of mesenchymal transition and stemness	36
S100A6	Astrocytic tumours	↑	doesn't show a significantly change in function of the level of tumour malignancy	49
	Ependymoma	↑	can be used to distinguish clinically and biologically relevant subgroups	50
S100A8	Glioblastoma	↑	presente a correlation with survival of GBM patients	66
	Glioma	↑	promote cell proliferation, invasion, and migration	69
S100A9	GSCs	↑	regulates GSCs proliferation	73
S100A8 and S100A9	Glioblastoma	↑	dependent on integrin signaling to promote migration and invasion at medium concentration	66
S100B	Glioma	↑	may be a valuable serum biomarker to predict the prognosis in glioma patients	84,85,86,87
	Astrocytoma	↑	contribute to astrocytomas progression by inhibiting p53 functions	92
S100P	Glioblastoma	↑	promote cell proliferation, migration, invasion and anchorage independent growth	94
S100A13	Astroglioma	↑	correlate with tumour grading and microvessel density	101
S100A11	Glioblastoma	↑	play crucial role in proliferation, EMT, migration, invasion and neurosphere formation	105,106

↑,Upregulation; ↓,Downregulation; -, Do not expression.
